# Sample Processing Methods Impacts on Rumen Microbiome

**DOI:** 10.3389/fmicb.2019.00861

**Published:** 2019-04-30

**Authors:** Gonzalo Martinez-Fernandez, Stuart E. Denman, Christopher S. McSweeney

**Affiliations:** Commonwealth Scientific and Industrial Research Organisation (CSIRO), Agriculture and Food, St Lucia, QLD, Australia

**Keywords:** rumen, microbial community, DNA, 16S sequencing, sample

## Abstract

The standardization of collection and processing methods for rumen samples is crucial to reduce the level of errors that may affect the analysis and interpretation of the data. The aim of this study was to compare two processing methods and their impacts on the microbial community composition analysis, from material that was either immediately frozen or samples that were stored as cell pellets after removing the supernatant prior to freezing. Eight rumen-fistulated Brahman steers received chloroform as an antimethanogenic compound for 21 days. Rumen fluid samples (60 mL per animal) were collected using a probe covered with two layers of cheesecloth at 3 h post feeding at day 0 prior-treatment (control period) and day 21 of treatment. One sub-set of samples were placed in dry ice and stored at −80°C (Method 1) for subsequent DNA extraction, while a second subset of samples was centrifuged, the supernatant removed and the microbial pellet and rumen contents placed in dry ice and stored at −80°C (Method 2) prior to DNA extractions. Phylogenetic based methods (Illumina Miseq) targeting the 16S rRNA gene were used to characterize the bacterial and archaeal communities from both collection methods for the control and treatment periods. The results from this study showed that the chloroform treatment was significantly different for all beta diversity measures regardless of the processing method used. Significant differences in the relative abundances of some bacteria and archaea, such as Elusimicrobia, Fibrobacteres, Lentisphaerae, Spirochaetes, and Verrucomicrobia and Methanomassiliicoccaceae, were observed at higher levels in the Method 2. These microbial populations are known to have fragile cell wall structures and are susceptible to cell lysis. Regardless of the processing method used, both identified the key microbial groups and can be used to compare the relative shifts in the rumen microbiome between treatments. However, immediately freezing samples might alter the abundance of material from species that are more readily lysed and will not be suitable for studies that aim to assign absolute abundance values to these species within the rumen.

## Introduction

The rumen microbiome is a very complex community formed by bacteria, fungi, protozoa, archaea and viruses. However, a low number of these microorganisms have been successfully isolated or are able to be cultured. In the last decades, the use of molecular biology tools have been essential to characterize this community, allowing the scientists to determine changes and functions within these microbial groups. Recently, in depth microbial ecology and metagenomic techniques have allowed for a high-resolution observation into the changes in rumen microbial populations with respect to relative abundance and level of metabolic activity within the system. However, the valuable information generated by these cutting edge tools is limited by a lack of standardized methods for collecting, processing and analyzing the samples, resulting in data which is often not comparable across studies.

An accurate understanding of the microbiome and its response to altering conditions within the rumen is crucial to design future strategies to manipulate it in regard to improvement of efficiency and decrease of the environmental impact from the host. The study of the microbiome is also crucial in any environmental system where it plays an important role, such us nitrogen fixation or marine systems. Thus, any finding in regard to the rumen microbiome might be used to understand other microbial ecosystems and vice versa.

Several studies have shown that the sampling technique (oral stomach tubing vs. rumen fistula) (Henderson et al., 2013; Ramos-Morales et al., 2014; Paz et al., 2016), sample processing (Bahl et al., 2012; McKain et al., 2013) and DNA extraction method (Henderson et al., 2013) can have a marked impact on the results. The standard procedures followed for collection of rumen fluid usually involve the storage of rumen aliquots at −20 to −80°C prior to extraction of nucleic material. However, little is known about the impact or biases that these processes may have on the results.

The aim of this study was to compare two processing methods and their impacts on the microbial analysis, from material that either was immediately frozen or samples that were stored as cell pellets after removing the supernatant prior to freezing. It was hypothesized that samples stored as cell pellets would show an increase in relative abundance of those bacterial groups with a more fragile cell wall compared to the samples immediately frozen.

## Materials and Methods

The experimental protocol complied with the Australian Code for the Care and Use of Animals for Scientific Purposes (8th Edition, 2013) and was approved by the local Animal Experimentation and Ethics Committee (A08/2014).

### Experimental Design and Sampling

Eight rumen-fistulated Brahman (*Bos indicus*) steers (body live weight, 493 ± 16 kg) were used in the experiment at Lansdown Research Station (Townsville, QLD, Australia). Animals received as diet forage *ad libitum* [Rhode grass hay (*Chloris gayana*)] and were adapted to the diet over an initial 21 days period. After that initial period, experimental animals were maintained in individual pens in an animal house (10 days) and were treated with cyclodextrin (2 g/100 kg LW). On day 10 rumen fluid was collected through the cannula (control period). Chloroform entrapped in cyclodextrin was used as antimethanogenic compound as described by Martinez-Fernandez et al. (2016). Following the control period animals received the chloroform-cyclodextrin (1.6 g/100 kg LW) through the cannula for another 21 days. On day 21 rumen fluid was collected as previously described.

Rumen fluid samples (60 mL per animal) were collected using a probe covered with two layers of cheesecloth at 3 h post feeding. One sub-set of samples (2 mL) were placed in dry ice and stored at −80°C (Method 1) for DNA extraction, while a second subset of samples (2 mL) was centrifuged (13,000×*g* for 5 min), the supernatant was removed and the microbial pellet was placed in dry ice and stored at −80°C (Method 2) prior to DNA extractions.

### DNA Extractions

The starting point for both methods was 2 mL of rumen fluid. DNA extractions were carried out on cell pellets (collection Methods 1 and 2) as previously described employing a bead beating method for lysis (Martinez-Fernandez et al., 2016) with minor modifications as follows: samples from Method 1 were centrifuged (13,000×*g* for 5 min), the supernatant was removed and the microbial pellet kept for DNA extraction. Cells from Method 1 and 2 were homogenized with 200 mg of silica-zirconium beads (1:1 mixture of 0.1 and 1.0-mm beads; Biospec, Bartlesville, OK, United States) and 800 μl of CTAB buffer in a Mini-Beadbeater-8 (Biospec) on maximum speed for 2 min, twice. Samples were incubated at 70°C for 20 min and centrifuged at 10,000×*g* for 10 min, and the supernatant was mixed with 500 μl of 25:24:1 phenol-chloroform-isoamyl alcohol (Fluka BioChemika, Buchs, Switzerland). The yield and purity of the extracted DNA were assessed with a NanoDrop 8000 spectrophotometer (Thermo Fisher Scientific, Wilmington, DE, United States).

### Real-Time PCR Analysis

The DNA samples from 4 animals were used as templates for quantifying the abundance of total bacteria 16rRNA gene, the *mcrA* gene for methanogens, and the 16S rRNA for Methanomassiliicoccaceae family specific. The primers and assay conditions used were previously published by Denman and McSweeney (2006), Denman et al. (2007), and Huang et al. (2016). Real-time PCR (qPCR) analyses were run in quadruplicate from one DNA extraction on an Applied Biosystems^TM^ ViiA^TM^ 7 Real-Time PCR System (Thermo Fisher Scientific Inc.,). Assays were set up using the SensiFAST SYBR^®^ Lo-ROX reagents (Bioline). Total rumen microbial DNA template concentration of 50 ng were used for each assay under the following cycle conditions: one cycle of 50°C for 10 s and 95°C for 2 min 30 s for initial denaturation, forty cycles at 95°C for 15 s and 60°C for 1 min for primer annealing and product elongation. Fluorescence detection was performed at the end of each annealing and extension step. Amplicon specificity was performed via dissociation curve analysis of PCR end products by raising the temperature at a rate of 0.05°C/s from 60 to 95°C. The fold change between Method 1 and Method 2 was calculated for each targeted population.

### 16S rRNA Analysis and Statistical Analyses

High throughput sequencing platforms, barcoding procedures for sample recognition and phylogenetic analysis of the 16S rRNA gene were used to characterize the microbial populations present in the rumen for the control and treatment periods. The V4 region of the 16S rRNA gene was targeted using specific primers (Kozich et al., 2013). Each individual DNA sample was amplified using the specific primers and a unique barcode combination. Afterward, amplification products were visualized by performing agarose gel electrophoresis. Product quantities were calculated and an equal molar amount of each product was pooled. The pooled products were run in a 1% agarose gel and bands were visualized and excised under blue light transillumination. The amplicons were gel purified with QIAquick Gel extraction Kit (Qiagen, Hilden, Germany) prior to submission for Illumina Miseq 250 bp paired-end (Macrogen Inc., South Korea).

Paired end short read sequence data generated on the Illumina Miseq was processed using the USEARCH package (Edgar, 2010). De-multiplexed paired end sequences were first merged prior to sequence quality filtering, followed by denoising (error correction) and chimera checking and clustering of sequences to operational taxonomic units (OTUs) of 97% similarity. Taxonomic assignment of sequences was performed against the Greengenes database (gg_13_5) (McDonald et al., 2012). An OTU table with counts per sample was generated using UPARSE and imported into Phyloseq R package (1.23.1) (McMurdie and Holmes, 2013). Alpha diversity measures for samples grouped by collection period and treatments were performed on unrarefied data using Physoleq. The OTU count data was log transformed as an approximate variance stabilization transformation process prior to beta diversity analysis. Beta diversity was performed as a principal coordinate analysis for the Bray Curtis dissimilarity distances and UniFrac for each sample using Phyloseq. The significances of grouping in the PCoA plots were tested by analysis of dissimilarity (ADONIS) with 999 permutations from the vegan package (Oksanen et al., 2013). Identification of OTUs significantly different between treatments represented as log2 fold changes for OTUs with adjusted *p*-values < 0.05 (false discovery rate)were calculated in the DESeq2 package (1.18.1) (Love et al., 2014). Plots were produced using the ggplot2 package (2.2.1) (Wickham, 2016). The effect of the method and treatment groups and their interaction were calculated for alpha diversity and changes in taxonomic group relative abundances, with the animal as the experimental unit using the linear mixed model from the lme4 package (Bates et al., 2015). The sequences obtained in this paper have been deposited in the European Nucleotide Archive under the accession number PRJEB31137.

## Results

Rumen samples collected from animals at control and chloroform treatment periods were stored at −80°C as rumen fluid (Method 1), or as a microbial cell pellet (Method 2). Both methods for sample processing produced high quality DNA (260/280: 1.93 and 1.91, respectively; *P* = 0.064; SEM = 0.006) with similar yields (466 vs. 412 ng DNA/μl; *P* = 0.187; SEM = 19.9) that were successfully used to amplify microbial 16S rRNA gene for microbial ecology analysis. The number of sequences obtained for each animal for the two sample processing methods was not different [χ^2^(1) = 0.037, *P* = 0.85] with a mean of 51083 and 50657, respectively.

Quantitative PCR analysis of the effect of method and treatment on the abundance of total bacteria (16rRNA gene), methanogens (*mcrA* gene) and Methanomassiliicoccaceae family are shown in Supplementary Table 1. The total bacteria abundance increased (2.36-fold) with Method 2 (*P* ≤ 0.001) compared with Method 1. A trend (*P* = 0.097) was observed for the Methanomassiliicoccaceae family, with 1.96-fold increase with Method 2. Only a treatment effect was observed for the *mcrA* gene (*P* = 0.031) and Methanomassiliicoccaceae family (*P* = 0.026), while the only treatment by method interaction was found for total bacteria.

**FIGURE 1:**
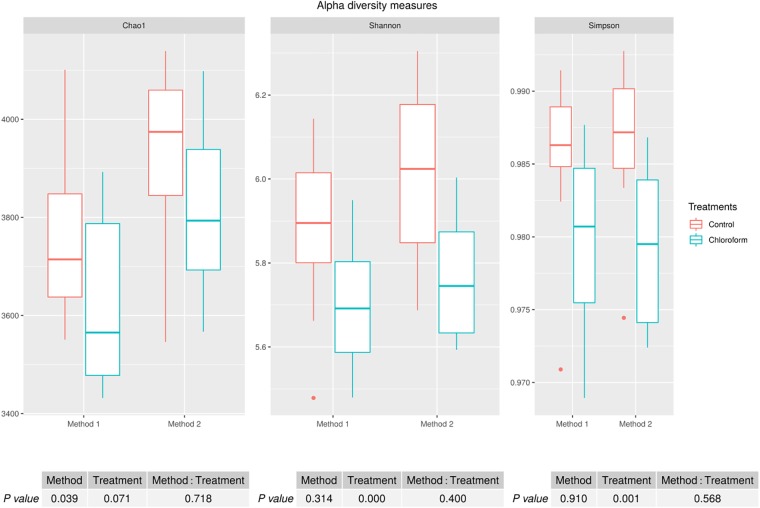
Alpha diversity measures for rumen microbiomes for control and chloroform treatments for processing Methods 1 and 2. Boxplots indicate variance within the sampled animals with the box boundaries showing the first and third quartiles, the median value indicated as a horizontal line and the whiskers extend to 1.5 times the interquartile range.

Alpha diversity measures for Chao1 estimates of the number of rumen microbiota OTU’s were significantly different between the methods (*P* = 0.039), with Method 2 on average detecting a further 170 OTUs (Figure 1). There was a non-significant decrease on average of approximately 120 OTUs for the chloroform treated animals compared to the controls regardless of the processing method (Figure 1). Although there was a difference between the methods for the Chao1 estimates, there was no method effect on the richness or evenness of the rumen microbiota as judged by Shannon and Simpson measures (Figure 1). Both methods identified significant decreases in diversity richness for the chloroform treatment as measured by the Shannon index (*P* < 0.001). Likewise, the evenness was significantly reduced due to the treatment for both methods as detected in the Simpson index (*P* = 0.001) (Figure 1). No significant interactions were detected within any diversity indexes.

**FIGURE 2:**
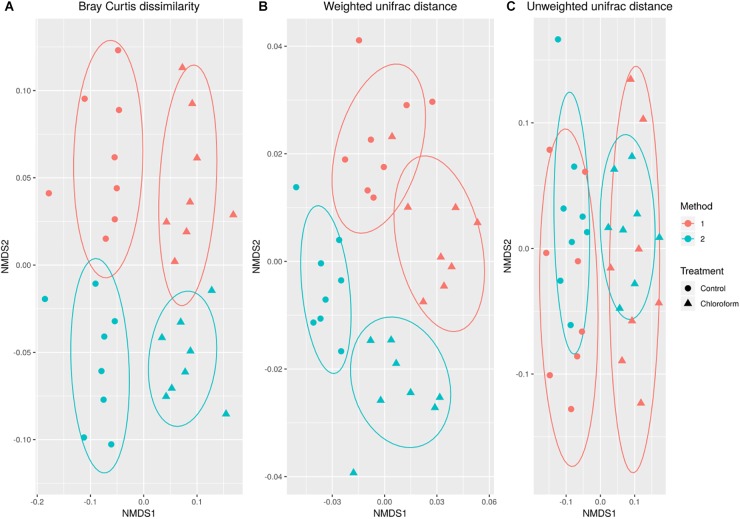
Non-metric MultiDimensional Scaling ordination comparing changes in microbial operational taxonomic unit (OTU) classification based on Bray-Curtis dissimilarity **(A)**, weighted **(B)** and unweighted **(C)** UniFrac calculations for control (•) and chloroform (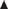) treatments at Method 1 (red) and Method 2 (light green).

Variance in the microbiota community structure as determined by beta diversity analysis was different between processing methods when abundance weights were included with either Bray Curtis or weighted UniFrac analysis (Figure 2). The extraction method explained 4 to 20% of the variance (weighted vs. unweighted), while the treatment explained 11 to 24% of the variance (Table 1). While ADONIS calculations on the centroids of unweighted UniFrac was significantly different for processing methods, the beta dispersions were also significantly different indicating heterogeneous dispersion between the samples was most likely influencing this effect. The chloroform treatment was significantly different for all beta diversity measures regardless of the processing method used.

**Table 1:** ADONIS for beta diversity distances comparing extraction methods and treatments.

	Method			Treatment		
	*R*^2^	*p*-value	Beta disp *p*	*R*^2^	*p*-value	Beta disp *p*
Bray Curtis	0.048	0.009	0.990	0.12	0.001	0.50
Weighted UniFrac	0.201	0.001	0.881	0.24	0.001	0.72
Unweighted UniFrac	0.042	0.025	0.049	0.11	0.001	0.95

**FIGURE 3:**
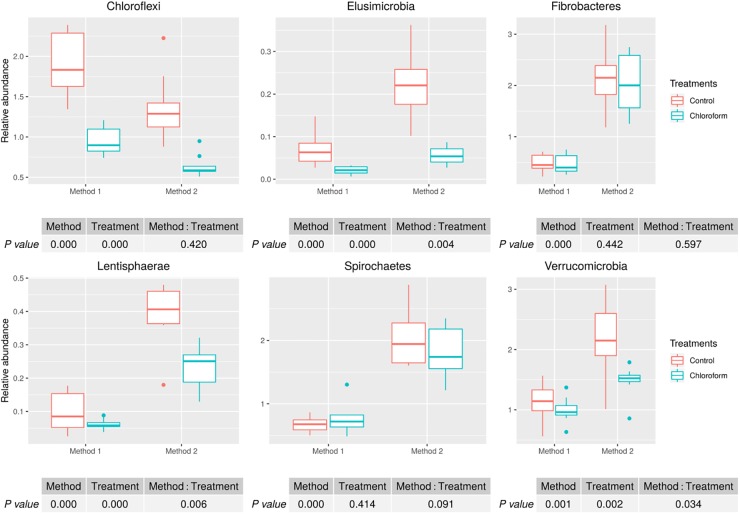
Microbial relative abundances (Chloroflexi, Elusimicrobia, Fibrobacteres, Lentisphaerae, Spirochaetes, and Verrucomicrobia) for treatment and method comparison.

**FIGURE 4:**
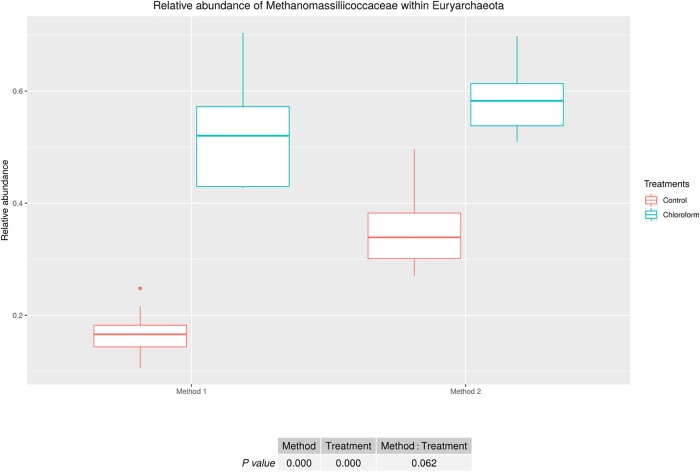
Methanomassiliicoccaceae relative abundance within Euryarchaeota for treatment and method comparison.

Analysis of the rumen microbiota showed changes within the phylum rank abundance order based on the processing method used (Supplementary Figure 1). Both methods were dominated by Bacteroidetes and Firmicutes and there was no significant (*P* = 0.803) change to the Firmicutes:Bacteroidetes ratio (Supplementary Figure 2). Method 2 resulted in an average 3-fold relative increase in detection of phyla assigned to Elusimicrobia, Fibrobacteres, Lentisphaerae, Spirochaetes, and Verrucomicrobia (Figure 3). The phylum Elusimicrobia, and the closely related Lentisphaerae and Verrucomicrobia, showed significant decreases in the relative abundance of these phylum in chloroform treated animals for Method 2, while Method 1 showed no significant change [Significant interaction effect for Lentisphaerae (*P* = 0.006), Verrucomicrobia (*P* = 0.034) and Elusimicrobia (*P* = 0.004)]. The Chloroflexi phylum was the only group significantly increased in Method 1, with a relatively minor 1.4-fold increase (*P* < 0.001) (Figure 3). Within the Euryarchaeota phyla, Method 2 detected increased relative abundances of Methanomassiliicoccaceae family compared to Method 1 (*P* < 0.001) (Figure 4). Both methods detected a significant difference in the relative abundance for the treatment effect (*P* < 0.001) with a trend in the interaction between the method and treatment (*P* = 0.062).

**FIGURE 5:**
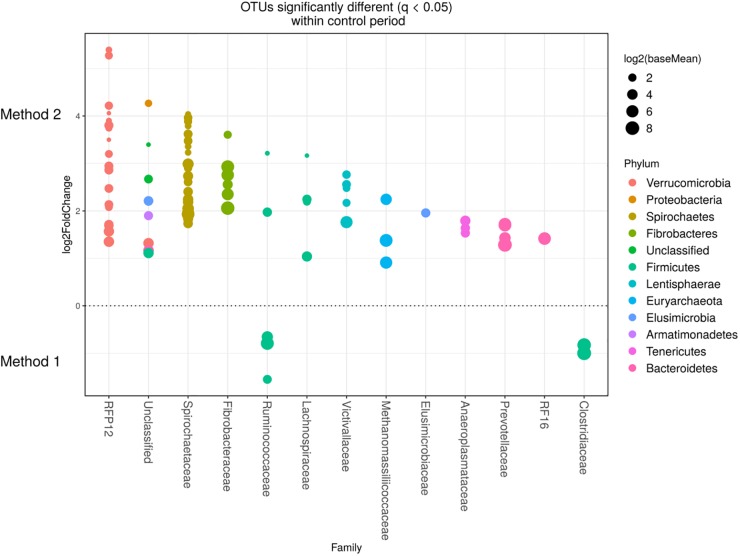
Operational taxonomic units significantly different (*q* < 0.05 FDR) between the two methods for the control period. Upper axis represents OTU’s with a log2 × fold positive difference for Method 2 relative to Method 1 while the lower *y* axis is the negative fold difference of the Method 2 relative to Method 1. Each point represents a single OTU colored by phylum and grouped on the *x* axis by taxonomic family level, size of point reflects the log2 mean abundance of the sequence data.

**FIGURE 6:**
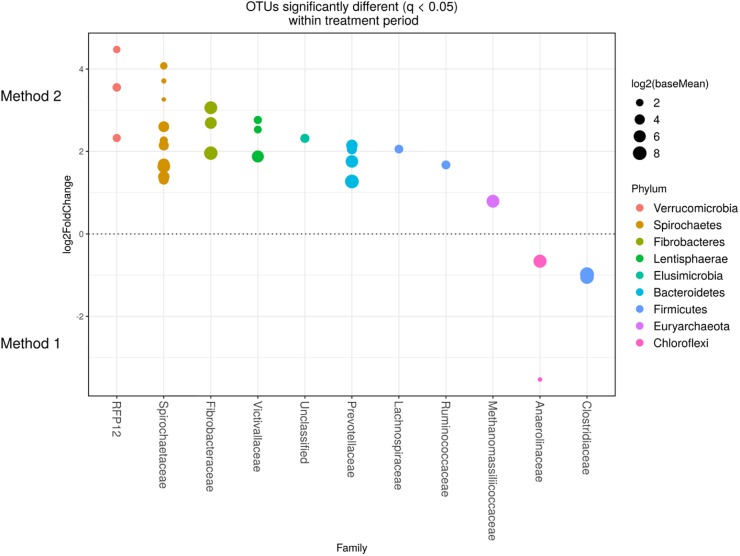
Operational taxonomic units significantly different (*q* < 0.05 FDR) between the two methods for the chloroform period. Upper axis represents OTU’s with a log2-fold positive difference for Method 2 relative to Method 1 while the lower *y* axis is the negative fold difference of the Method 2 relative to Method 1. Each point represents a single OTU colored by phylum and grouped on the *x* axis by taxonomic family level, size of point reflects the log2 mean abundance of the sequence data.

**FIGURE 7:**
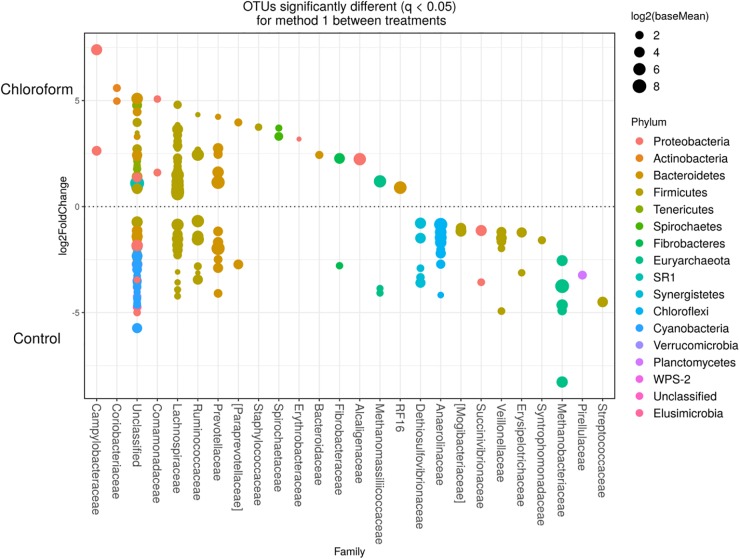
Operational taxonomic units significantly different (*q* < 0.05 FDR) between chloroform treated-animals and control for Method 1. Upper axis represents OTU’s with a log2 fold positive difference for chloroform treatment relative to control while the lower *y* axis is the negative fold difference of the chloroform relative to control. Each point represents a single OTU colored by phylum and grouped on the *x* axis by taxonomic family level, size of point reflects the log2 mean abundance of the sequence data.

**FIGURE 8:**
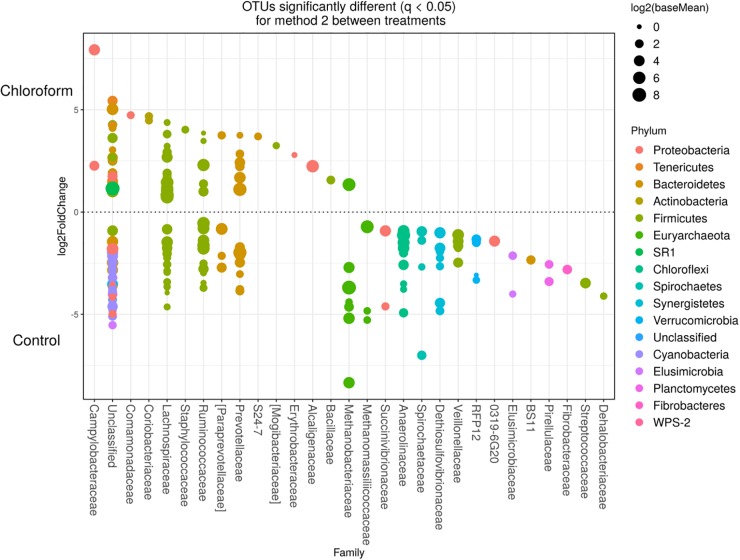
Operational taxonomic units significantly different (*q* < 0.05 FDR) between chloroform treated-animals and control for Method 2. Upper axis represents OTU’s with a log2 fold positive difference for chloroform treatment relative to control while the lower *y* axis is the negative fold difference of the chloroform relative to control. Each point represents a single OTU colored by phylum and grouped on the *x* axis by taxonomic family level, size of point reflects the log2 mean abundance of the sequence data.

Detected variance in specific OTUs based on the processing methods used, aligned with the above observed changes at the phylum level. Method 2 showed increases in relative abundance of OTUs assigned mainly to the families RFP12, Spirochaetaceae, Fibrobacteraceae, Lachnospiraceae, Victivallaceae, Prevotellaceae, Methanomassiliicoccaceae, and Anaeroplasmataceae, compared with Method 1 at both the control or chloroform treatment periods (*q* < 0.05) (Figure 5, 6). Only OTUs within the Anaerolineaceae, Clostridiaceae, and Ruminococaeeae families were increased with Method 1. No significant interaction effect was found between the treatments and methods for any OTU (*q* > 0.05), indicating that the same population changes were detected regardless of the processing method used. Similar compositional shifts in specific OTUs between the chloroform treated animals and the control period with both methods were observed (Figure 7, 8). Chloroform treatment of animals resulted in decreased Euryarchaeota, predominately from the Methanobacteriaceae family, along with decreased Synergistetes and Verrucomicrobia.

## Discussion

The standardization of collection and processing methods for rumen samples is crucial to reduce the level of unintentional errors that may affect the analysis and interpretation of the data. Henderson et al. (2013) have highlighted the effect varying DNA extraction methods has on the rumen microbial community composition that is detected, limiting the capacity for comparison when different extraction methods have been used across studies.

Many DNA extraction methodologies for rumen samples, begin with stored frozen material, followed by centrifugation of thawed samples and removal of the supernatant and addition of lysis/extraction buffers. Cells lysed during the freezing stage may release DNA from their cells which would be lost with the removal of the rumen fluid supernatant, reducing their relative abundance. Immediately centrifuging fresh rumen samples and removing the supernatant prior to freeze storage was assessed in this study as a way to minimize this hypothesized effect.

Only a few papers have addressed the effects that sample storage post collection may have on microbial compositional analysis. The ratio of the Gram positive Firmicutes to Gram negative Bacteroidetes has been suggested as a biomarker for defining gut microbial community composition, so minimizing sample processing errors is important (Costea et al., 2018). Fliegerova et al. (2014) compared the rumen bacterial profile of DNA extracted from the cell-free supernatant and microbial pellet, finding a lower proportion of Firmicutes in the supernatant sample and similar proportion of Bacteroidetes in both samples. Freezing human stool samples for storage resulted in a significantly higher Firmicutes to Bacteroidetes ratio compared to fresh samples (Bahl et al., 2012). Likewise, rumen samples that were frozen without glycerol as a cryoprotectant also produced increased ratios of Firmicutes to Bacteroidetes (McKain et al., 2013). No differences in this ratio were observed in this study and may reflect the high relative abundance of Bacteroidetes in these cattle compared to other studies. However, increases in the relative abundance of less abundant Gram negative bacterial groups, were observed when using DNA from Method 2. Suggesting that directly freezing the rumen sample increases the extent of Gram negative bacteria cell lysis. Gram positive and Gram negative bacteria differ in their cell wall structure, in particular the thickness of the peptidoglycan layers that surround the membrane (Silhavy et al., 2010). The peptidoglycan constituent of the bacteria cell wall, is distributed in layers and is between 10 and 100 times thicker in Gram positive than Gram negative bacteria. Interestingly, Bahl et al. (2012) suggested that the increase in Firmicutes was possibly due to the improved extraction or stability of DNA from Gram positive bacteria because of the freezing process. However, the opposite would also hold for our Method 1 process, in which freezing could lyse a proportion of Gram negative bacteria and allow removal of DNA prior to extraction during the initial centrifugation step to remove the supernatant component of the sample. In fact, an increase (2.36-fold) in total bacteria using qPCR was observed in Method 2, which support our hypothesis.

In the present study, a wide range of Gram negative bacteria, classified within the Spirochaetes, Fibrobacteres and Verrucomicrobia amongst others phyla, were detected at increased levels in Method 2 compared with the first Method. The Spirochaetes phylum, has a fragile outer envelope, making them sensitive to adverse conditions (Johnson, 1977). *Fibrobacter succinogenes*, which is a member of the Fibrobacteres phylum, presents straight-chain fatty acids in its membrane with no sphingolipids (Ransom-Jones et al., 2012), which might make them more susceptible to lysis. The phylum Verrucomicrobia has a complex cell structure with a fragile intracytoplasmic membrane (Hedlund, 2010) which differentiates them from other bacteria and might explain our observation. Other minor Gram negative groups that also increased with Method 2, were assigned to Lentisphaerae, Armatimonadetes and Elusimicrobia. The Lentisphaerae phylum is closely related to Verrucomicrobia and shares a similar intracellular structural plan (Gupta et al., 2012), which might explain the increase. Regarding Elusimicrobia, some species have a thin membrane (Zheng et al., 2015) due to their small size, which might increase their fragility.

The relative abundance of total Euryarchaeota were not affected by the processing methods, although members of the Methanomassiliicoccaceae family were relatively more abundant in samples processed using Method 2. The lower relative abundance observed in the immediately frozen samples (Method 1) compared with Method 2, is likely due to the unique characteristics of the cell wall/membrane in this group of methanogens, which does not contain pseudomurein. In fact, Borrel et al. (2014) reported that the components of cell wall/membrane and envelope biogenesis were less abundant in the order Methanomassiliicoccales than in other gastrointestinal methanogens. Dridi et al. (2012), found a single thin electron-dense layer and one thick transparent layer in *Methanomassiliicoccus luminyensis* isolated from human feces, which differs from the thicker electron-dense layer from other methanogens. In addition, other members of the order Thermoplasmata, present in other environments, do not present with cell walls and instead have a thin layer cell membrane (Itoh et al., 2007). The quantitative results support the sequencing data, showing a trend of 1.96-fold increase for the Methanomassiliicoccaceae family in Method 2.

Irrespective of the processing method used the same microbial diversity was still detected as indicated through un-weighted beta diversity analysis. Yet weighted analysis showed clear variance between the methods to be linked to the abundance of those OTUs. Regardless of the differences observed between the processing methods, a similar shift in specific OTUs was reported between the treatment and control periods for both methods. Indicating that the same population changes were obtained regardless of the processing methods used. The changes observed were in accordance with published studies using the same antimethanogenic compound (Martinez-Fernandez et al., 2016, 2017), mainly with compositional shifts within Bacteroidetes and Firmicutes populations and a decrease in relative abundance of OTUs belonging to the Euryarchaeota, Synergistes, and Verrucomicrobia phyla.

## Conclusion

In conclusion, Method 2 increased the relative abundance of Gram negative populations as a proportion of sequencing effort from rumen liquid samples. However, when performing relative fold changes between “treatments” the extraction method did not affect the detection of populations that have altered their relative abundance. Nevertheless, immediately freezing rumen samples altered the abundance of DNA material from species that were more readily lysed and might not be suitable for studies that aim to assign abundance values to these species within the rumen. Further analyses are needed to confirm that the concentration of these species is lower in the immediately frozen samples.

## Ethics Statement

The experimental protocol complied with the Australian Code for the Care and Use of Animals for Scientific Purposes (8th Edition, 2013) and was approved by the local Animal Experimentation and Ethics Committee (A08/2014).

## Author Contributions

CM, SD, and GM-F conceived and designed the experiments and analytical approaches and wrote the manuscript. GM-F performed the animal trial and analyzed the biological samples. SD analyzed the data. All authors agreed to be accountable for all aspects of the work.

## Conflict of Interest Statement

The authors declare that the research was conducted in the absence of any commercial or financial relationships that could be construed as a potential conflict of interest.
