# Supplementary material for: Sample Processing Methods Impacts on Rumen Microbiome
Source: Front Microbiol. 2019 Apr 30;10:861. doi: 10.3389/fmicb.2019.00861 (PMC6502991; doi:10.3389/fmicb.2019.00861)
Supplement: Supplementary file 1 [file Data_Sheet_1.docx]

***Supplementary Material***

**Sample processing methods impacts on rumen microbiome**

**Gonzalo Martinez-Fernandez^*^, Stuart E. Denman and Christopher S. McSweeney**

CSIRO, Agriculture and Food, St Lucia, QLD, Australia

*** Correspondence:** Gonzalo Martinez Fernandez, CSIRO, Agriculture and Food, Queensland Bioscience Precinct, 306 Carmody Road, St Lucia, QLD, 4067, Australia. Email:gonzalo.martinezfernandez@csiro.au

|  | Treatment | |  |  | *P-value* | | |
| --- | --- | --- | --- | --- | --- | --- | --- |
|  | Control | Chloroform | SEM |  | Treatment | Method | T x M |
| Total bacteria (16S) | 3.50 | 1.21 | 0.08 |  | 0.513 | 0.001 | 0.003 |
| Methanogens (*mcrA*) | 0.90 | 1.55 | 0.12 |  | 0.031 | 0.746 | 0.368 |
| Methanomassiliicoccaceae family (16S) | 2.18 | 1.73 | 0.25 |  | 0.026 | 0.097 | 0.534 |

**Supplementary Table 1.** Fold change of Method 2 compared with Method 1 of total bacteria 16rRNA gene, methanogens *mcrA* gene and Methanomassiliicoccaceae family population by quantitative PCR. The method, treatment and its interaction were analyzed for each gene. Analysis was performed in samples from 4 animals.


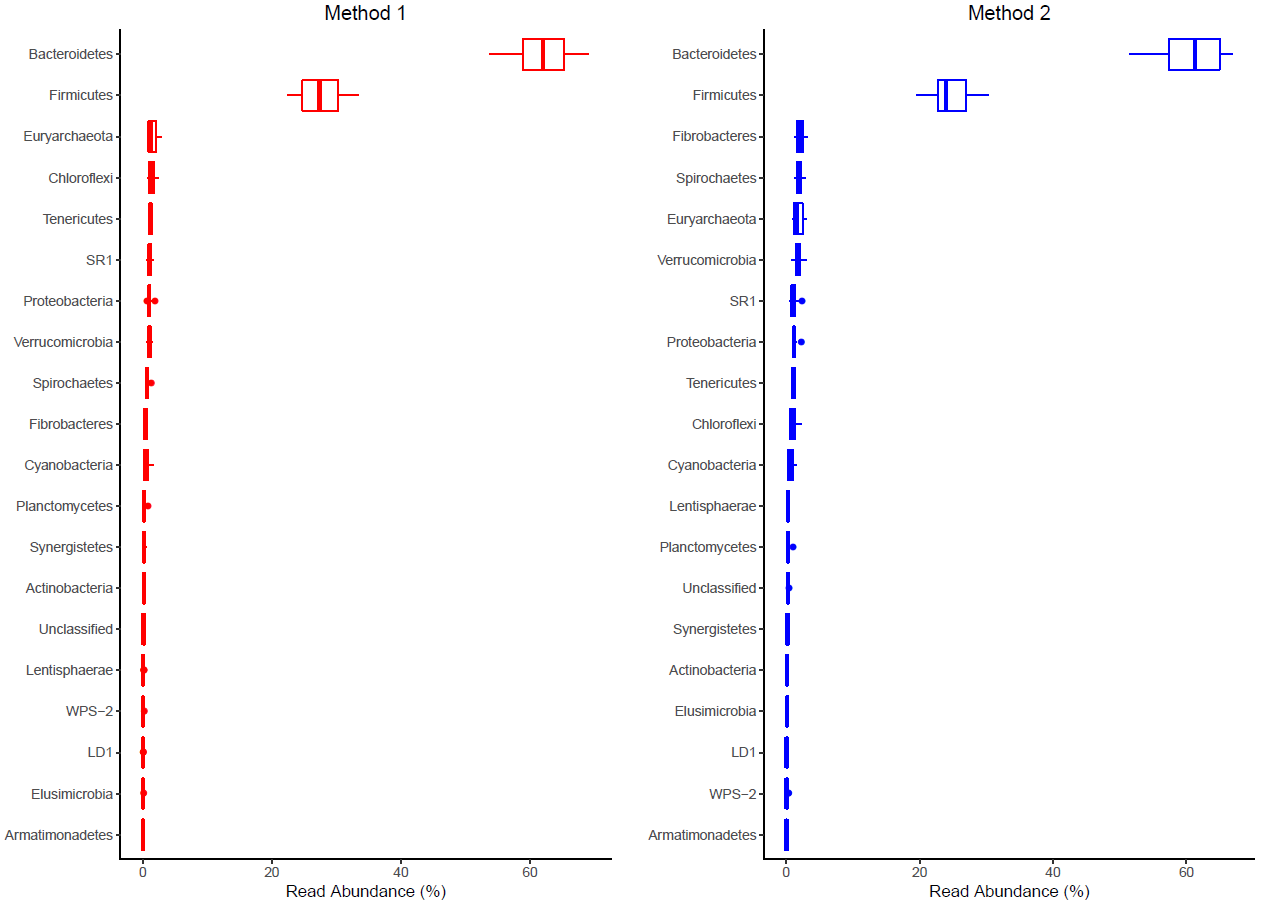


**Supplementary Figure 1.** Phylum level relative rank abundance plots for samples processed using either method 1 (left) or method 2 (right).


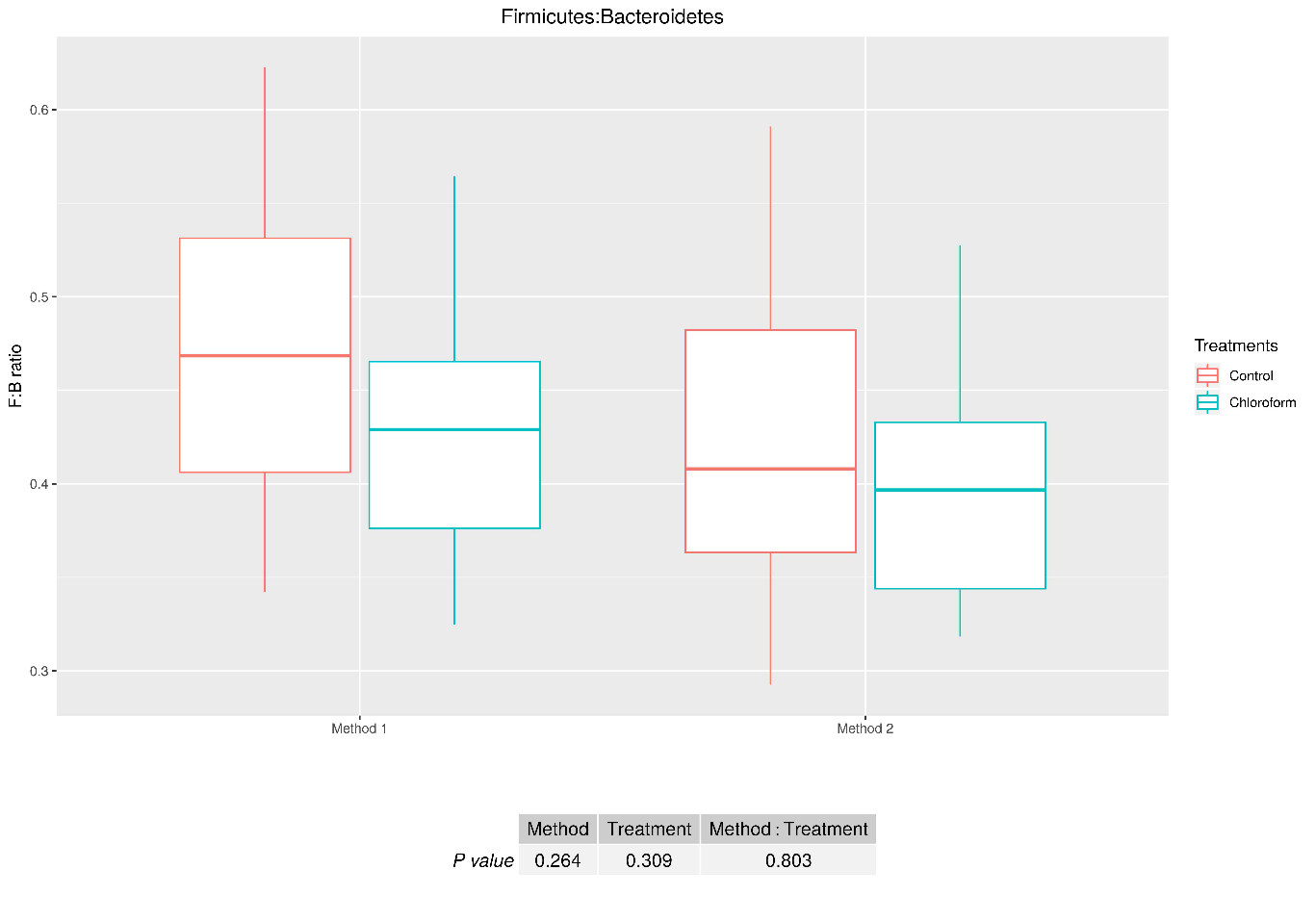
**Supplementary Figure 2.** Firmicutes to Bacteroidetes ratio for treatment and method comparison.
